# The relationship between sleep quality and physical learning engagement among Chinese college students: a variable centered and person centered analysis

**DOI:** 10.3389/fpubh.2026.1845627

**Published:** 2026-05-05

**Authors:** Shiyu Zhang, Rongting Liu, Fang Rao, Qiong Zhou, Qun Xiong

**Affiliations:** 1 Physical Education College, Shanghai University of Sport, Shanghai, China; 2 Ji’an College, Ji’an, Jiangxi, China; 3 Department of Physical Education, Science and Technology College of NCHU, Jiujiang, Jiangxi, China; 4 Physical Education College, Jiangxi Normal University, Jiujiang, Jiangxi, China; 5 Department of Rehabilitation II, Affiliated Hospital of Jiangxi University of Traditional Chinese Medicine, Nanchang, Jiangxi, China

**Keywords:** college students, latent profile analysis, physical learning engagement, self esteem, serial mediation model, sleep quality, smartphone addiction

## Abstract

**Objective:**

This study examined the association between sleep quality and physical learning engagement among Chinese college students and tested the serial mediating roles of smartphone addiction and self-esteem. Both variable centered and person centered approaches were adopted to elucidate the underlying mechanisms.

**Methods:**

A total of 1,130 college students completed the Pittsburgh Sleep Quality Index (PSQI), the Utrecht Work Engagement Scale for Students (UWES-S), the Mobile Phone Addiction Index (MPAI), and the Rosenberg Self-Esteem Scale (SES). Descriptive statistics, correlation analyses, regression analyses, and mediation analyses were conducted in SPSS 26.0 using the PROCESS macro. Latent profile analysis (LPA) was performed in Mplus 8.3.

**Results:**

Sleep quality was negatively associated with physical learning engagement (*β* = −0.096, *p* < 0.05). Smartphone addiction partially mediated the association between sleep quality and physical learning engagement (indirect effect = −0.145, 95% CI [−0.258, −0.040]). Self esteem also showed a significant partial mediating effect (indirect effect = −0.131, 95% CI [−0.213, −0.065]); In addition, smartphone addiction and self-esteem jointly constituted a significant serial indirect association between sleep quality and physical learning engagement (indirect effect = −0.027, 95% CI [−0.049, −0.010]). LPA further identified four subgroups based on smartphone addiction and self esteem, and these latent profiles differed significantly in sleep quality and physical learning engagement.

**Conclusion:**

The findings showed that poorer sleep quality was significantly associated with lower vigor, dedication, and absorption in physical learning engagement. This association operated through three pathways, a single mediating effect of smartphone addiction, a single mediating effect of SE, and a serial mediating pathway via smartphone addiction and then self esteem, although the overall effect sizes were small. Latent profile analysis further identified four heterogeneous subgroups based on joint levels of smartphone addiction and self esteem; among them, the high-addiction–moderate-self esteem group exhibited the poorest sleep quality and the lowest physical learning engagement. These results provide an empirical basis for stratified interventions in higher education and suggest that improving students’ sleep and regulating excessive smartphone use may be effective strategies to enhance physical learning engagement.

## 1 Introduction

Sleep problems among college students have become increasingly prevalent and are now recognized as a major public health concern in higher education worldwide. Sleep is not only essential for physiological restoration but also plays a critical role in memory consolidation, sustained attention, emotion regulation, and executive functioning (1, 2). College students commonly experience insufficient sleep (3, 4), difficulty initiating sleep, and circadian rhythm disruption (5, 6). Approximately 20 to 40% of college students sleep less than the recommended 7 to 9 h for their age group (7–9), and many report that sleep is among the first health behaviors to be sacrificed during university life (10). Prior studies have shown that poor sleep quality (SQ) is highly prevalent in this population, with 30 to 70% of students classified as poor sleepers (11). Evidence has also indicated that COVID-19-related restrictions significantly disrupted students’ sleep patterns, including bedtime schedules, sleep latency, and sleep duration, resulting in marked deterioration in SQ (12). In China, the 2022 *Sleep White Paper* reported that 75% of the population experience sleep disturbances, and that young adults aged 19–25 years sleep only 6.46 h on average (13), suggesting an especially high prevalence of sleep-related problems in this age group (14). Poor SQ has also been linked to lower grade point average and poorer academic performance (15). Taken together, these findings suggest that SQ is not merely an indicator of physical and psychological well-being, but also an important antecedent of academic engagement and adjustment among college students.

**Figure 1:**
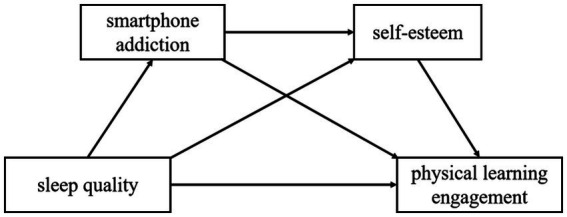
The hypothesized model diagram.

The importance of physical education in higher education has been widely acknowledged (16). High-quality physical education not only enhances students’ exercise attitudes and motor skills, but also contributes to physical health, social competence, emotional regulation, and social responsibility (17–19). Within this context, physical learning engagement (PLE) is particularly important because it directly associated with the effectiveness of physical education. Low PLE may lead to poor motor skill acquisition, negative learning attitudes, and insufficient participation in physical activity (20, 21). Such outcomes may, in turn, adversely affect students’ physical and mental health, academic functioning (22), and even their long-term quality of life (23). PLE refers to the positive psychological state and behavioral involvement students’ display in physical education classes, typically characterized by vigor, dedication, and absorption. Unlike more sedentary academic subjects, physical learning is highly embodied, skill-based, and interaction-dependent, requiring sustained attention, bodily coordination, and emotion regulation. Investigating how SQ is associated with PLE may help clarify the specific effects of sleep deprivation on embodied learning and may also provide practical guidance for health promotion in university physical education. Accordingly, we proposed the following hypothesis (see Figure 1):

*Hypothesis 1*: SQ is significantly associated with PLE among college students.

SQ is a central factor influencing college students’ physical and psychological health and daily functioning (24). Beyond its direct relevance to physiological recovery and cognitive maintenance (25), SQ may also shape digital behavior patterns and psychological resources, thereby affecting PLE. Sleep insufficiency and poor SQ have been shown to impair prefrontal executive functioning (26), weaken emotion regulation and self-evaluation systems (27), and increase dependence on immediately rewarding behaviors such as smartphone use (28). At the same time, diminished self-worth and lower SE resulting from sleep deprivation (15), may undermine students’ intrinsic motivation to participate in physically demanding and skill-challenging activities (29). Mobile phone addiction has been linked to insomnia and poorer sleep-related outcomes in university samples, and smartphone addiction has been shown to play a chain-mediating role between physical activity and SQ via stress (30). Other studies have reported that parental psychological control is related to higher levels of mobile phone addiction, with physical activity moderating this association (31), and that different types or levels of physical activity are associated with differences in mobile phone addiction or craving for smartphone use (32, 33). On this basis, we proposed the following hypothesis (see Figure 1):

*Hypothesis 2*: SA mediates the relationship between SQ and PLE.

From a psychological perspective, self-esteem (SE) may also be closely related to the association between SQ and PLE. SQ has been linked to emotion regulation, mood, and self-evaluative processes (34), and college students with more sleep problems tend to report higher levels of negative affect and psychological distress, as well as less favorable self-views (35–37). At the same time, SE is widely regarded as a core psychological resource that is associated with students’ motivation, persistence, and willingness to engage with challenging academic and physical tasks. Higher SE has been related to more positive achievement beliefs and greater engagement in learning activities, whereas lower SE has been associated with avoidance, performance anxiety, and reduced participation in physical education contexts (38, 39). Ke et al. (40) further reported that SE statistically mediated the association between physical activity and smartphone addiction among Chinese college students, indicating that SE may function as an intervening psychological characteristic in the link between health-related behaviors and problematic smartphone use. Similarly, SE has been shown to mediate the association between physical activity and internet addiction and to relate robustly to mental health indicators and adjustment in higher education settings (41). Thus, we further hypothesized that (see Figure 1):

*Hypothesis 3*: SE mediates the relationship between SQ and PLE.

Poor SQ and smartphone addiction (SA) are both common among college students (42, 43). From both behavioral and psychological perspectives, SA and self esteem (SE) may represent key mediating mechanisms linking SQ to PLE. On the one hand, students with poor SQ may prolong smartphone use to cope with daytime fatigue or to fill periods of nocturnal wakefulness, and such behavior may gradually develop into compulsive smartphone dependence (44, 45). Moreover, the blue-light exposure, cognitive arousal, and social comparison associated with excessive smartphone use may further exacerbate sleep problems, thereby creating a vicious cycle (46). On the other hand, sleep deprivation may impair emotion regulation and self-evaluation, potentially lowering SE, whereas individuals with lower SE may be more likely to seek external validation through virtual social interaction, thereby increasing their vulnerability to SA (47, 48). Previous studies have documented the serial mediating roles of SA and SE in the association between physical activity and sleep quality; however, to our knowledge, this mechanism has not yet been extended to the specific academic context of PLE (47). On this basis, we proposed a serial mediation hypothesis (see Figure 1):

*Hypothesis 4*: SA and SE jointly exert a serial mediating effect in the relationship between SQ and PLE.

Most previous studies have used a variable centered approach to examine associations among variables (49). Although this approach is useful for identifying general patterns, it may overlook important heterogeneity in how psychological and behavioral characteristics cluster within individuals (50). College students may differ substantially in their combinations of SA and SE, and these differences may be associated with distinct patterns of SQ and PLE. Thus, a variable centered perspective alone may not be sufficient to fully capture the mechanisms through which SQ affects PLE. A person centered approach, such as latent profile analysis (LPA), can identify meaningful subgroups based on psychological and behavioral configurations, thereby providing more nuanced evidence regarding which students are at greater risk and offering a basis for stratified intervention and precision management in university settings.

Against this background, the present study integrated health behavior and social cognitive perspectives to examine the association between SQ and PLE among Chinese college students, while testing the independent and serial mediating roles of SA and SE. In addition, LPA was used to explore heterogeneity in the combined characteristics of SA and SE and to compare SQ and PLE across latent subgroups. By integrating variable centered and person centered approaches, this study aimed to provide a more comprehensive account of the mechanism linking sleep status, digital behavior, psychological resources, and learning performance, and to generate theoretical and practical implications for sleep health education, digital behavior intervention, and psychological support in higher education.

## 2 Materials and methods

### 2.1 Participants

An *a priori* power analysis was conducted in G*Power 3.1 based on a regression-based mediation model. Assuming a medium effect size (*f*^2^ = 0.15), a significance level of *α* = 0.05, and statistical power of 1 − *β* = 0.95 (51), the minimum required sample size was estimated to be 119. In addition, conventional guidelines in psychology and behavioral research recommend a sample size of at least 10 times the number of questionnaire items (52). Given that the questionnaire used in this study comprised 63 items in total, the minimum sample size according to this criterion was 630.

A cluster sampling method was used to recruit college students from two universities in Hunan Province, China. Informed consent was obtained from all participants prior to data collection. The survey was conducted on January 13, 2026, using both online and paper-and-pencil questionnaires administered during the first 15 min of physical education classes. A total of 1,254 questionnaires were distributed. Responses were excluded if they contained missing data, exhibited patterned responding, or had a completion rate below 75%. After data screening, 1,130 valid questionnaires were retained, yielding an effective response rate of 90.11%. Participants ranged in age from 18 to 23 years, with a mean age of 19.67 ± 1.15 years.

### 2.2 Measures

#### 2.2.1 Sleep quality

SQ was assessed using the Pittsburgh Sleep Quality Index (PSQI), a widely used self-report instrument designed to evaluate overall sleep quality during the previous month (53). The PSQI contains 19 self-rated items and yields seven component scores (e.g., “Have you often felt tired in the past month”), each ranging from 0 to 3, with higher scores indicating poorer sleep quality. The global score ranges from 0 to 21. The internal consistency reliability was satisfactory, with a Cronbach’s *α* of 0.734 (54).

#### 2.2.2 Physical learning engagement

PLE was measured using the Utrecht Work Engagement Scale-Student (UWES-S) developed by Schaufeli et al. (55). The scale conceptualizes engagement in terms of three dimensions: vigor, dedication, and absorption. It includes 17 items (e.g., “I feel full of energy during physical education class”) rated on a 5-point Likert scale ranging from 1 (“strongly disagree”) to 5 (“strongly agree”). The internal consistency reliability was satisfactory, with a Cronbach’s *α* of 0.913 (56).

#### 2.2.3 Smartphone addiction

SA was assessed using the Mobile Phone Addiction Index (MPAI), developed by Leung (57). The scale comprises four dimensions and 17 items (e.g., “I find myself spending more time on my phone than I intended to”), each rated on a 5-point scale. Higher scores indicate a greater tendency toward SA. The internal consistency reliability was satisfactory, with a Cronbach’s *α* of 0.870 (58).

#### 2.2.4 Self esteem

SE was measured using the Rosenberg Self-Esteem Scale (SES), a widely used instrument assessing general feelings of self-worth and self-acceptance (59). The scale contains 10 items (e.g., “I can do things just as well as most people”) and has demonstrated satisfactory factorial and criterion validity. The internal consistency reliability was satisfactory, with a Cronbach’s *α* of 0.780 (60).

### 2.3 Data analysis

Data were analyzed in SPSS 26.0 using descriptive statistics, correlation analyses, and regression analyses. Mediation analyses were conducted using Hayes’ PROCESS macro, and the significance of indirect effects was tested using bias-corrected percentile Bootstrap confidence intervals. Model 6 in PROCESS was used to test the serial mediation model. Given the cross-sectional design, these analyses are exploratory and test statistical associations rather than causal mechanisms.

To further examine heterogeneity in SA and SE from a person centered perspective, LPA was conducted in Mplus 8.3. Item-level indicators from the SA and SE measures were used as manifest variables, and models with an increasing number of latent classes were estimated sequentially. Model fit was evaluated using the Akaike Information Criterion (AIC), Bayesian Information Criterion (BIC), sample-size-adjusted Bayesian Information Criterion (aBIC), Lo–Mendell–Rubin test (LMR), Bootstrapped Likelihood Ratio Test (BLRT), and entropy. Lower AIC, BIC, and aBIC values indicate better fit; significant LMR and BLRT values indicate that the current model fits better than the model with one fewer class; and entropy values closer to one indicate greater classification accuracy (61). The optimal model was selected based on fit indices, class proportions, classification quality, and theoretical interpretability. One way analysis of variance was then conducted to compare SQ and PLE across latent classes, followed by *post hoc* comparisons.

## 3 Results

### 3.1 Common method bias and normality assessment

To assess the potential impact of common method bias (CMB), we conducted a confirmatory factor analysis (CFA) by specifying a single-factor model in which all measurement items were loaded onto one common latent factor. The results indicated a poor model fit: CFI = 0.438, RMSEA = 0.142, and SRMR = 0.195, all of which fall well outside the commonly accepted thresholds (CFI < 0.90, RMSEA > 0.08, SRMR > 0.08) (62). In addition, Harman’s single-factor test was used to assess common method bias, and an unrotated principal component analysis was performed on all questionnaire items (63). Eight factors had eigenvalues greater than 1, and the first factor accounted for 27.16% of the total variance, which was below the conventional threshold of 40%. Taken together, the results from both approaches provide convergent evidence that common method bias is not a serious concern in this study. Normality was then evaluated. As reported in Table 1, the skewness and kurtosis values indicated that the variables did not deviate substantially from normality (i.e., skewness < 3.0 and kurtosis < 10.0) (64).

**Table 1:** Normality assessment of the data.

Normality Statistics	SQ	PLE	SA	SE
Skewness	0.904	−0.695	0.541	0.261
Kurtosis	0.990	0.634	−0.173	0.481

SA, smartphone addiction; PLE, physical learning engagement; SE, self-esteem; SQ, sleep quality; the same as below.

### 3.2 Descriptive statistics and correlation analysis

As presented in Table 2, PLE was significantly negatively correlated with SQ (*r* = −0.142, *p <* 0.01), SA (*r* = −0.161, *p* < 0.01), and SE (*r* = −0.167, *p <* 0.01). In addition, SQ was significantly positively correlated with SA (*r* = 0.320, *p <* 0.01) and SE (*r* = 0.228, *p* < 0.01), and SA was also significantly positively correlated with SE (*r* = 0.178, *p* < 0.01).

**Table 2:** Correlation analysis results.

Variable	*M*	SD	PLE	SQ	SA	SE
PLE	63.12	15.55	1			
SQ	4.48	3.17	−0.142^**^	1		
SA	35.95	14.56	−0.161^**^	0.320^**^	1	
SE	24.45	5.51	−0.167^**^	0.228^**^	0.178^**^	1

**p < 0.01.

### 3.3 Regression analysis

As shown in Table 3, When SA is included as a dependent variable, SQ is positively correlated with SA after controlling for gender and age (*β* = 0.317, *p <* 0.001), and gender was also significantly associated with SA (*β* = 0.129, *p <* 0.001). The model was statistically significant (*F* = 50.880, *p <* 0.001) and explained 11.9% of the variance (*R*^2^ = 0.119). When SE was regressed on gender, age, SQ, and SA, the overall model was significant (*F* = 22.276, *p <* 0.001) and accounted for 7.3% of the variance (*R*^2^ = 0.073), reflecting a small effect size. SQ (*β* = 0.199, *p* < 0.001) and SA (*β* = 0.128, *p* < 0.001) were both positively correlated with SE, whereas gender was negatively correlated with SE (*β* = −0.086, *p* = 0.003). When PLE was treated as the dependent variable and gender, age, SQ, SA, and SE were entered simultaneously, the regression model was significant (*F* = 18.780, *p <* 0.001) and explained 7.7% of the variance (*R*^2^ = 0.077). SQ (*β* = −0.096, *p =* 0.002), SA (*β* = −0.093, *p =* 0.003), and SE (*β* = −0.134, *p <* 0.001) were all significantly negatively correlated with PLE, but the effect size is relatively small. In addition, gender (*β* = −0.116, *p <* 0.001) and age (*β* = 0.124, *p <* 0.001) were also significantly associated with PLE. Although several pathways reached statistical significance, the corresponding effect sizes were relatively small, suggesting that these associations should be interpreted with caution and do not imply causal relationships.

**Table 3:** Regression analysis among variables.

Regression equation		Overall model fit			Significance of regression coefficients		
Outcome variable	Predictor variable	*R*	*R* ^2^	*F*	*β*	*t*	*p*
SA	Gender	0.346	0.119	50.880	0.129	4.602	0.000^**^
	Age				−0.034	−1.178	0.239
	SQ				0.317	11.163	0.000^**^
SE	Gender	0.271	0.073	22.276	−0.086	−2.962	0.003^*^
	Age				−0.040	−1.359	0.174
	SQ				0.199	6.482	0.000^**^
	SA				0.128	4.181	0.000^**^
PLE	Gender	0.278	0.077	18.780	−0.116	−3.996	0.000^**^
	Age				0.124	4.258	0.000^**^
	SQ				−0.096	−3.058	0.002^*^
	SA				−0.093	−3.015	0.003^*^
	SE				−0.134	−4.506	0.000^**^

*p < 0.05, **p < 0.001.

### 3.4 Mediation analysis

**Figure 2:**
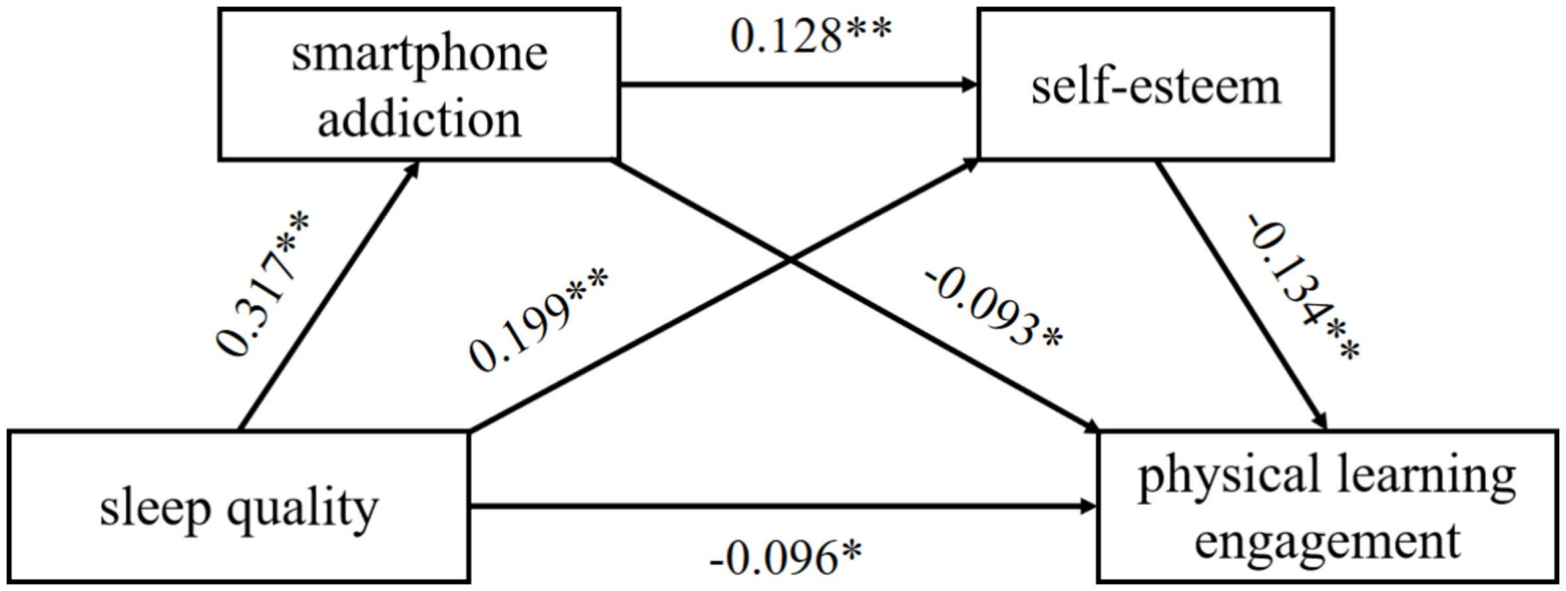
Chain mediation model diagram.

After controlling for gender and age, the serial mediating roles of SA and SE in the association between SQ and PLE were tested. The total effect of SQ on PLE was significant (*β* = −0.302, 95% CI [−0.429, −0.184]). All three indirect pathways were significant. First, the indirect effect through SA alone was −0.145 (95% CI [−0.258, −0.040]), accounting for 47.78% of the total effect. Second, the indirect effect through SE alone was −0.131 (95% CI [−0.213, −0.065]), accounting for 43.39% of the total effect. Third, the serial indirect effect through SA and SE was −0.027 (95% CI [−0.049, −0.010]), accounting for 8.83% of the total effect. These findings supported the proposed serial pathway from SQ to PLE via SA and SE. Comparisons among the indirect effects indicated no significant difference between the SA pathway and the SE pathway, whereas both single-mediator pathways were significantly stronger than the serial mediation pathway (see Table 4 and Figure 2).

**Table 4:** Test results of bootstrap mediation effect.

Path	Effect	BootSE	BootLLCI	BootULCI	Proportion
Total	−0.302	0.062	−0.429	−0.184	100.00%
Ind1: SQ → SA → PLE	−0.145	0.055	−0.258	−0.040	47.78%
Ind2: SQ → SE → PLE	−0.131	0.038	−0.213	−0.065	43.39%
Ind3: SQ → SA → SE → PLE	−0.027	0.010	−0.049	−0.010	8.83%
C1: Ind1-Ind2	−0.013	0.073	−0.156	0.133	—
C2: Ind1–Ind3	−0.118	0.058	−0.232	−0.008	—
C3; Ind1–Ind3	−0.105	0.035	−0.183	−0.046	—

### 3.5 Person centered analysis

**Figure 3:**
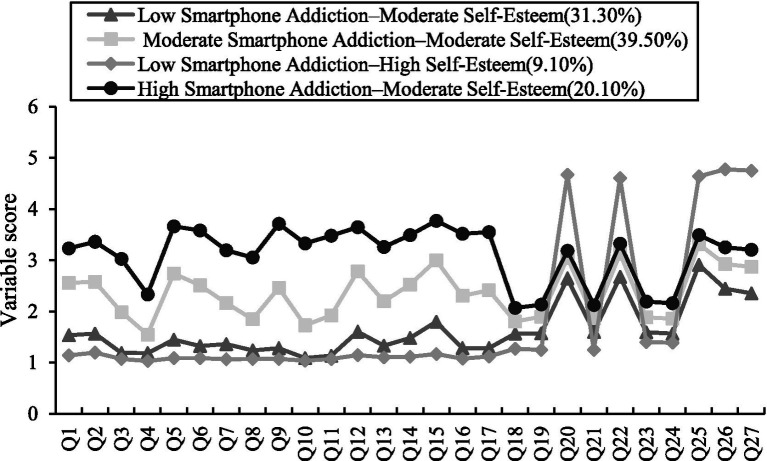
Distribution of characteristics across four potential categories of SA and SE.

LPA was conducted using item-level indicators of SA and SE. As the number of classes increased, AIC, BIC, and aBIC values progressively decreased. The LMR test was significant for the two-class through five class solutions but not for the six class solution (*p* = 0.6814), whereas the BLRT remained significant across all models. The four-class model showed adequate fit, significant LMR and BLRT results (*p <* 0.001), an entropy value of 0.940, and class proportions of 31.30, 39.50, 9.10, and 20.10%, respectively. According to the recommended guidelines for potential profile analysis (65), Considering model fit, classification accuracy, class distribution, and theoretical interpretability, the four class solution was selected as the optimal model (see Table 5). Based on the mean score distributions, the four latent classes were labeled as low addiction–moderate SE, moderate addiction–moderate SE, low addiction–high SE, and high addiction–moderate SE (see Figure 3). This labeling approach follows established conventions in person-centered research, whereby profiles are named according to their relative standing (e.g., low, moderate, high) on key indicators to enhance interpretability and comparability across studies (50). These findings suggest substantial heterogeneity in the combined characteristics of SA and SE among college students. One-way ANOVA showed significant differences across the four latent classes in both SQ (*F* = 50.451) and PLE (*F* = 12.050). *Post hoc* comparisons indicated that the high addiction–moderate SE group had the poorest sleep quality, whereas the low addiction–moderate SE group had the best sleep quality. In terms of PLE, the low addiction–moderate SE group had the highest scores, whereas the high addiction–moderate SE group had the lowest scores (See Table 6).

**Table 5:** Fit indices for latent profile models.

Model	AIC	BIC	aBIC	LMR (*p*)	BLRT (*p*)	Entropy	Categorical probability %
Class 1	88756.665	89028.283	88856.764				
Class 2	81139.966	81552.424	81291.969	<0.0001	<0.0001	0.939	59.98%/40.02%
Class 3	78565.057	79118.354	78768.963	<0.0001	<0.0001	0.938	33.13%/43.28%/23.59%
Class 4	76689.170	77383.306	76944.979	<0.0001	<0.0001	0.940	31.30%/39.50%/9.10%/20.10%
Class 5	75448.886	76283.862	75756.598	0.0077	<0.0001	0.944	32.87%/8.78%/13.19%/29.47%/15.69%
Class 6	74307.328	75283.143	74666.943	0.6814	<0.0001	0.941	13.85%/22.24%/30.32%/7.15%/11.63%/14.81%

**Table 6:** Differences in SQ and PLE across latent profiles.

Variable	A1 (356)	A2 (445)	A3 (103)	A4 (226)	*F*	Back texting
SQ	3.03 ± 2.43	4.96 ± 2.86	4.23 ± 3.67	5.95 ± 3.57	50.451	A4 > A2 > A3 > A1
PEE	66.57 ± 15.04	61.92 ± 13.99	64.74 ± 21.63	59.27 ± 14.85	12.050	A1 > A3 > A2 > A4

## 4 Discussion

### 4.1 Direct association between sleep quality and physical learning engagement

This study found that SQ was significantly negatively associated with PLE, such that poorer SQ was linked to lower vigor, dedication, and absorption in physical education classes. This finding supports Hypothesis 1 and is consistent with previous evidence that sleep deprivation undermines cognitive functioning and academic performance (66). Ng et al. (67) demonstrated that poorer sleep quality undermines academic performance by reducing academic engagement among university students. Similarly, Suardiaz-Muro et al. (68) found a significant positive association between sleep quality and self-reported academic achievement during the examination period. From a neurocognitive perspective, insufficient sleep impairs prefrontal executive control, including sustained attention, inhibitory control, and goal-directed behavior (69). Because physical learning is an embodied form of learning that requires active physical participation, skill execution, and contextual interaction, it places high demands on students’ attentional control, bodily coordination, and emotion regulation. When these capacities are compromised by poor sleep, students may find it more difficult to maintain concentration, overcome physical fatigue, and engage actively in classroom tasks. In addition, sleep deprivation may disrupt memory consolidation by affecting hippocampal functioning (70). Because motor skill acquisition relies heavily on procedural memory consolidation during sleep, poor SQ may impede the retention of newly learned physical skills, thereby reducing learning efficiency and diminishing motivation and engagement in physical education classes.

### 4.2 Mediating role of smartphone addiction

The results supported Hypothesis 2 by showing that SA partially mediated the association between SQ and PLE. Poor SQ was associated with higher SA, which in turn was associated with lower PLE. This pathway is consistent with recent findings that smartphone addiction is closely related to sleep problems and insomnia among Chinese college students, often via complex mediated or moderated mechanisms (71). It suggests that students with poorer sleep may use smartphones excessively to cope with fatigue or fill wakeful time at night, and such behavior may gradually develop into compulsive dependence (72). Smartphone applications provide immediate, frequent, and low-effort reinforcement through notifications, likes, and game rewards, thereby strengthening habitual use (73). Once excessive smartphone use consumes time and cognitive resources that might otherwise be devoted to physical participation and classroom learning, PLE is likely to decline. Moreover, SA and poor sleep may reinforce one another in a cyclical manner (74). Poor sleep weakens cognitive control and increases susceptibility to immediately rewarding behaviors, whereas bedtime smartphone use may further delay sleep onset and worsen SQ through blue-light exposure, cognitive arousal, and social comparison (75). In line with this, Wang et al. (30) showed that smartphone addiction plays a key mediating role in the link between stress and sleep quality among Chinese college students, highlighting the importance of addressing digital behavior when targeting sleep-related problems. Taken together with evidence that physical activity can mitigate mobile phone addiction and related health risks (76). These findings suggest that interventions targeting sleep problems should also address problematic digital behavior.

### 4.3 Mediating role of self esteem

Hypothesis 3 was also supported, although the direction of the SE pathway differed from the original expectation. Specifically, SQ and SA were positively associated with SE, whereas SE was negatively associated with PLE. At face value, this pattern appears counterintuitive, because prior work has often linked poor sleep and problematic smartphone use with lower SE, and higher SE with greater engagement in health-related behaviors, including physical activity and sport participation (40). One possible explanation for this pattern is that contemporary college students are chronically exposed to high levels of academic competition, interpersonal comparison, and uncertainty about the future. Under such pressure, the formation and expression of self-esteem may become highly contextualized and defensive (77). With respect to the link between sleep and self-esteem, students with poorer sleep quality tend to show impaired emotion regulation and reduced stress tolerance, and may therefore rely more on self-protective positive evaluations to maintain psychological balance (78). In practice, this can manifest as deliberately accentuating self-affirming aspects and downplaying weaknesses in self-reports, thereby producing higher scores on self-esteem scales (79). Such self-esteem is not grounded in genuine improvements in competence, but may instead represent a defensive self-enhancement strategy used to cope with sleep deprivation and psychological fatigue. In addition, College students, are frequently exposed to considerable anxiety, social comparison, and environmental pressure (35, 37), which may complicate the way SE relates to both digital behavior and physical learning.

Traditional theories suggest that sleep deprivation weakens emotion regulation and self-evaluation, thereby lowering SE (36). A possible additional explanation in our study is that students with higher SE do not necessarily perceive themselves as competent or efficacious in the domain of physical learning. For example, some students may feel generally confident in social or academic domains but have a low physical self-concept or low perceived motor competence (80). Despite relatively high SE, when these students are required to engage in structured physical learning tasks, the mismatch between their global self-worth and perceived physical ability may reduce their willingness to invest effort, thereby leading to lower PLE. This interpretation is consistent with research emphasizing the importance of perceived physical competence and domain-specific self-concept for predicting participation and persistence in sport and physical activity (81).

### 4.4 Serial mediating roles of smartphone addiction and self-esteem

The findings further supported Hypothesis 4, demonstrating a significant serial mediation pathway from SQ to smartphone addiction (32), then to SE, and finally to PLE (82, 83). Although the magnitude of this indirect effect was smaller than those of the two single-mediator pathways, Its statistical significance suggests that sleep is associated with PLE through a continuous chain involving both behavioral and psychological processes. Poor SQ may first impair cognitive control and emotion regulation, thereby increasing dependence on smartphones. Although excessive reliance on virtual social interactions and immediate feedback may elevate levels of self-esteem, it may also undermine motivation to engage in physically demanding and skill-oriented learning activities. This serial pathway underscores the interconnected nature of sleep status, digital behavior, psychological resources, and learning performance.

The comparison of indirect effects further showed that the mediating effects of SA and SE alone were each significantly stronger than the serial mediation effect. This suggests that the behavioral pathway and the psychological pathway may each play a relatively prominent role in the association between SQ and PLE, whereas their joint transmission chain, although meaningful, contributes less strongly. One possible explanation is that SA and SE may be bidirectionally correlated. This interactive relationship to some extent weakens the effect value of chain mediation (84). From a practical standpoint, this pattern suggests that interventions may target either SA or SE as key leverage points, while integrated interventions may be preferable when broader or more sustained effects are desired. At the same time, the relatively weaker serial mediation implies that the process from SQ to PLE is not simply a linear progression from sleep to smartphone behavior to self-evaluative processes, but may involve overlapping mechanisms and feedback loops between SA and SE (85). Sleep problems may simultaneously impair emotion regulation and increase reliance on smartphones, thereby influencing SE and learning engagement through partly parallel routes rather than a single, ordered chain (86). Future longitudinal studies with multidimensional assessments of SE and repeated measures of SA are needed to clarify temporal ordering and potential reciprocal relations between these variables.

### 4.5 Person centered findings from latent profile analysis

The variable centered analyses revealed a relatively stable pattern in which SQ was associated with PLE both directly and indirectly via smartphone addiction and SE. However, these analyses did not capture heterogeneity across individuals. The LPA addressed this issue by identifying four distinct subgroups based on SA and SE. The existence of these latent classes indicates that college students are not simply distributed along a single continuum of “higher addiction and lower SE,” but instead exhibit more complex psychological-behavioral configurations. In this sense, the person centered findings complement the variable centered model by showing that, although SA and SE are linked at the aggregate level, their configurations differ meaningfully across individuals.

The comparison of latent classes further showed that the high addiction–moderate SE group had the poorest SQ and the lowest PLE, whereas the low addiction–moderate SE group showed the most favorable profile. This finding highlights the value of a person centered approach in revealing the heterogeneous impact of combined digital-behavioral and psychological profiles on learning adjustment. Previous research has similarly reported heterogeneity in nighttime-specific smartphone use and SA among college students, with distinct subgroups differing in bedtime procrastination and sleep disturbance (63). Moreover, a growing body of evidence has consistently linked SA to poorer SQ in university populations (47, 87, 88).

Notably, the low addiction–high SE group did not show the highest level of PLE and in fact performed less favorably than the low addiction–moderate SE group. This finding suggests that the association between SE and PLE may not be linearly positive. From a situational adaptation perspective, physical learning requires sustained physical effort, motor skill practice, and tolerance for temporary failure. Students with excessively high self-esteem may be overly sensitive to performance outcomes and avoid intensive engagement to prevent potential frustration or self-doubt (89). In contrast, students with moderate self-esteem tend to hold more realistic self-perceptions, focus on task progress rather than self-image protection, and thus maintain more stable and active participation in physical learning (90). This explains why moderate SE, rather than high SE, correlates with better PLE when smartphone addiction is well controlled. Under such circumstances, a positive self-evaluation may not translate into greater willingness to embrace challenge or persist in effortful physical learning tasks. On the contrary, in physical education settings that involve public performance, bodily exposure, and competence comparison, these students may reduce engagement to avoid threats to self-image. Thus, what appears to be most beneficial for PLE may not be high SE per se, but rather a combination of lower smartphone dependence and more stable, internalized self-worth.

From a theoretical perspective, individuals may turn to online activities to cope with stress, fatigue, or low satisfaction in real life, thereby gradually developing addictive patterns that further undermine sleep and engagement (91). It is consistent with the Interaction of Person-Affect-Cognition-Execution model, which conceptualizes addictive behaviors as the product of interactions among personal traits, affective responses, cognitive control, and executive functioning (92). Prior research has further shown that SA may harm sleep through reduced self-regulation, bedtime procrastination, negative emotions, and perceived stress (93, 94). These findings suggest that universities may benefit from implementing stratified interventions based on latent subgroup membership, particularly for students in high-risk profiles. Such interventions should combine sleep hygiene education, digital behavior management, and efforts to foster more stable sources of self-worth.

## 5 Limitations of the study and future prospects

This study has several limitations. First, its cross-sectional design precludes causal inference; the mediation models should be interpreted as describing statistical associations rather than causal processes. Future research could incorporate longitudinal designs, cross-lagged models, or intervention studies to further examine the temporal dynamics and directional relationships among these variables. Second, all variables were measured via self-report at a single time point, which may have introduced social desirability bias, recall bias, and shared method variance; although anonymity and validated instruments were used, common method bias cannot be fully excluded. Future research should incorporate multi-source and more objective indicators. Third, participants were recruited from two universities in Hunan Province, which may limit the generalizability of the findings to other regions, institutional types, or cultural contexts. Fourth, the explanatory power and effect size of the models are relatively small, indicating that SQ, SA, and SE account for only a limited proportion of variance and that other unmeasured factors are likely important. The practical implications of the present findings should therefore be viewed as preliminary and not overstated. Fifth, the present study relied on a global measure of self-esteem, which constrains the conclusions that can be drawn about specific forms of SE (e.g., contingent, fragile, or externally based self-worth). Future research should employ more fine-grained assessments of SE to directly test whether these facets account for the seemingly counterintuitive associations observed in the current model.

## 6 Conclusion

This study surveyed 1,130 Chinese college students and integrated variable-centered and person-centered approaches to examine the association between SQ and PLE, as well as its underlying mechanisms. The findings showed that poorer SQ was significantly associated with lower vigor, dedication, and absorption in PLE. This association operated through three pathways, a single mediating effect of SA, a single mediating effect of SE, and a serial mediating pathway via SA and then SE, although the overall effect sizes were small. Latent profile analysis further identified four heterogeneous subgroups based on joint levels of SA and SE; among them, the high-addiction–moderate-SE group exhibited the poorest SQ and the lowest PLE. These results provide an empirical basis for stratified interventions in higher education and suggest that improving students’ sleep and regulating excessive smartphone use may be effective strategies to enhance PLE.

## Data Availability

The original contributions presented in the study are included in the article/supplementary material, further inquiries can be directed to the corresponding author.
